# Lactobacillus elicits a 'Marmite effect' on the chicken cecal microbiome

**DOI:** 10.1038/s41522-018-0070-5

**Published:** 2018-11-09

**Authors:** Angela Zou, Shayan Sharif, John Parkinson

**Affiliations:** 1 Program in Molecular Medicine, Hospital for Sick Children, Peter Gilgan Center for Research and Learning, 686 Bay Street, Toronto, ON M5G 0A4 Canada; 2 Department of Biochemistry, University of Toronto, Toronto, ON Canada; 3 Department of Pathobiology, Ontario Veterinary College, University of Guelph, Guelph, ON N1G 2W1 Canada; 4 Department of Molecular Genetics, University of Toronto, Toronto, ON Canada

## Abstract

The poultry industry has traditionally relied on the use of antibiotic growth promoters (AGPs) to improve production efficiency and minimize infection. With the recent drive to eliminate the use of AGPs, novel alternatives are urgently required. Recently attention has turned to the use of synthetic communities that may be used to ‘seed’ the developing microbiome. The current challenge is identifying keystone taxa whose influences in the gut can be leveraged for probiotic development. To help define such taxa we present a meta-analysis of 16S rRNA surveys of 1572 cecal microbiomes generated from 19 studies. Accounting for experimental biases, consistent with previous studies, we find that AGP exposure can result in reduced microbiome diversity. Network community analysis defines groups of taxa that form stable clusters and further reveals *Lactobacillus* to elicit a polarizing effect on the cecal microbiome, exhibiting relatively equal numbers of positive and negative interactions with other taxa. Our identification of stable taxonomic associations provides a valuable framework for developing effective microbial consortia as alternatives to AGPs.

## Introduction

The association of antibiotic growth promoter (AGPs) usage with antimicrobial resistance is prompting the poultry industry to seek alternative feed supplements.^1^ AGPs are used to increase production efficiency and reduce flock infections.^1^ While their precise mode of action is not known, AGPs are thought to work through altering the microbial community (microbiome) in the livestock gastrointestinal tract.^2^ Currently, interest lies in finding combinations of previously identified probiotics that can be used to promote the development of a healthy microbiome. To better understand stably associating taxa, we present a meta-analysis of published 16S rRNA surveys of the chicken ceca to identify key interactions/influencers in the chicken cecal microbiome. Previous publications have reported microbiome responses under a variety of conditions; including the effects of feed additives, *Eimeria* challenge, and breeding conditions. However, experimental biases of individual studies have led to conflicting results, especially when investigating the effects of AGPs.^3^ By combining datasets, it may be possible to discern general patterns of microbiome behaviour that are consistently found across all studies.

## Results and discussion

### Limitations of technical biases on microbiome meta-analyses

**Fig. 1:**
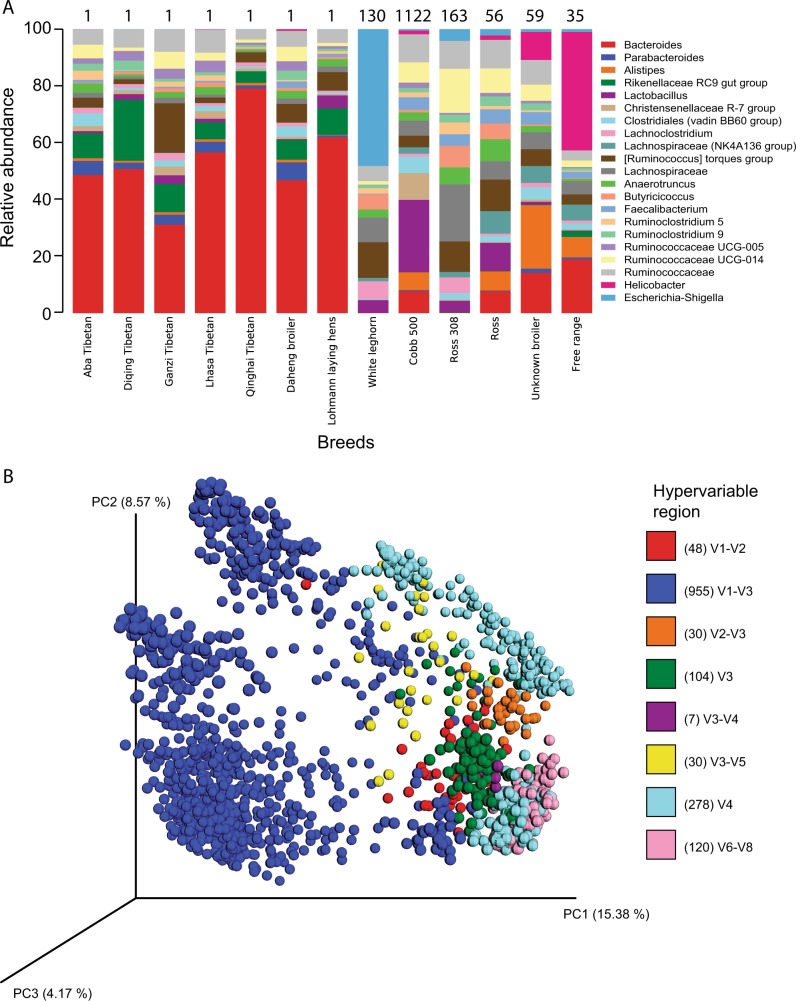
Microbial diversity of 1572 cecal samples from chicken. **a** Relative abundance of the most abundant genera by chicken breeds. Number on top of bars represent the number of sequencing samples for each breed, note that certain samples are pooled from multiple chicken cecal samples (see supplementary table 1). Only taxa present at greater than 1% were included. **b** Principal-coordinate analysis plot of unweighted UniFrac distances coloured according to hypervariable region. Numbers in brackets are the number of samples sequenced using each hypervariable region

16S rRNA gene sequences from 1572 chicken cecal samples were collated from 19 studies (Supplementary Table 1). We assigned ~22 million 16S rRNA gene sequences to 3300 OTUs (See Supplemental Information). Consistent with previous studies,^4^ *Bacteroidetes, Firmicutes*, and *Proteobacteria* were the dominant phyla, with relative proportions varying by breed (Fig. 1a and Supplementary Fig. 1). Relative to other breeds, broilers from commercial primary breeders, Cobb and Ross, exhibited similar profiles albeit Cobb exhibited a higher proportion of *Christensenellaceae* and *Lactobacillus*. Of the two layers included in this study (White leghorn and Lohmann), the microbiome profile of commercial Lohmann layers closely resembled the profiles of Chinese Tibetan chicken samples, which were sequenced and extracted by the same study, potentially reflecting study bias. Indeed, PCoA revealed that microbiome structure segregated by individual studies (Fig. 1b, Supplementary Fig. 2), suggesting they may be influenced by technical biases present, similar to the results of other microbiome meta-analyses.^5,6^

Moreover, sequencing region strongly influenced alpha diversity comparisons; we observed that AGP-treated samples sequenced using the V4, V3, and V6-V8 hypervariable regions exhibited significantly higher diversity (*t*-test; *p*-value < 0.05) than non-AGP-treated samples, most of which were sequenced by V1-V3 and 454 Roche (Supplementary Fig. 3). However, after partitioning data based on the region of the 16S rRNA gene targeted for sequencing, AGP-treated samples consistently display equal or lower diversity compared to control groups regardless of hypervariable region used (Supplementary Fig. 4, Supplementary Table 2), consistent with previous studies. Given that different regions of the 16S rRNA gene vary in length and sequence diversity,^7^ it is not unexpected that phylogenetic resolutions and subsequent within-diversity analysis were also found to differ for each region (Supplementary Fig. 3). Furthermore, sequencing platforms differ in error rates and sequencing depth, both of which were found to impact the number of OTUs detected within a sample (Supplementary Fig. 3). This is consistent with findings from other meta-analyses^5,8^ and highlights the need to be cautious when interpreting results from 16S rRNA-based meta-analyses, particularly when datasets may be generated using different methodologies.

### Co-occurrence network identifies unstable microbial clusters

**Fig. 2:**
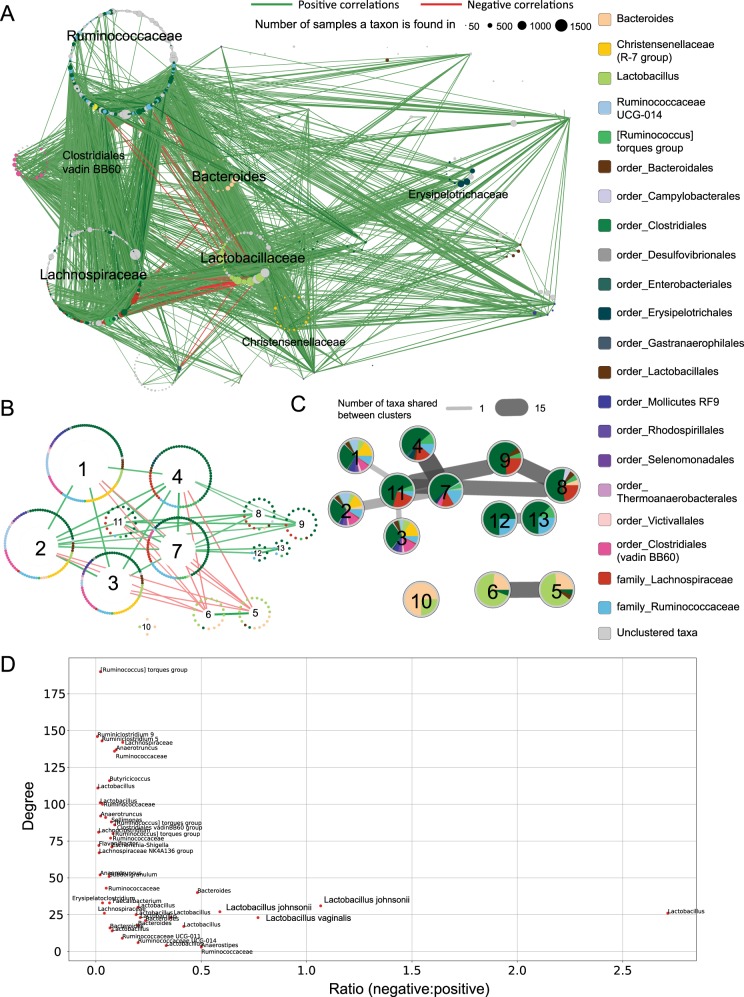
Co-occurrence network and analysis of OTUs chicken cecal samples. **a** Co-occurrence network built with SparCC with nodes representing taxa (as defined by OTUs—see Methods) and edges representing positive (green) or negative (red) associations of co-occurrence across samples. Thickness and opacity of the edges represent the strength of the correlation and node sizes represent the number of samples that contain those taxa. Taxa are grouped by family, with major families labelled. Correlations with an absolute value smaller than 0.3 are not shown. Colour of nodes indicate taxon (see legend), taxa that could not be resolved at the level of genus are noted with preceding *order* or *family*. **b** Clustered co-occurrence network with only the interactions between clusters shown. Nodes, representing taxa, are organized into a circular layout according to cluster membership. Each cluster is assigned a number for reference. **c** Number of taxa shared across clusters. Here each cluster is depicted as a pie chart with sectors indicating proportion of each taxon. Cluster numbering is consistent with (**b**). Edges between clusters indicate that there are taxa shared between clusters, thicker and darker edges represent more shared taxa. **d** Scatter plot of ratio of negative to positive interactions against degree for every taxon. Taxonomic labels down to the species level were obtained from sequence similarity searches against partitions of the NCBI’s non-redundant nucleotide database (see Supplemental Information)

To identify groups of microbes that co-exist in natural communities, we constructed a network of taxonomic associations (See Supplemental Information). In general, we found that *Lactobacillus* strains are negatively correlated with *Ruminococcaceae* and *Lachnospiraceae* strains, and instead form positive associations with other *Lactobacilli*, *Bacteroides* and *Christensenellaceae* (Fig. 2a). Moreover, the network is scale-free (Supplementary Fig. 5), i.e., the network is dominated by a limited number of taxa exhibiting a large number of connections that have a major influence on community structure, together with large numbers of taxa with relatively few connections. To define groups of well-connected microbes, we clustered taxa based on patterns of co-occurrence (Figs. 2b, c). Two clusters (clusters 5 and 6) were largely composed of *Lactobacillus* strains together with a more restricted set of *Bacteroides, Ruminococcaceae*, and unclassified *Bacteroidales*. Interestingly, both clusters exhibited negative correlations with several clusters dominated by *Clostridiales* (clusters 1, 2, 3, 4, 7 11). These negative associations may reflect the presence of members of *Mollicutes, Ruminococcaceae UCG-014, Clostridiales (vadinBB6), and Christensenellaceae R-7 group*, which are absent in the four other *Clostridiales*-dominated groups (clusters 8, 9, 12 and 13) with which no negative associations were observed.

Previous studies have suggested that microbiomes may be classified into *enterotypes* based on the co-occurrence of discrete groups of taxa.^9^ We therefore attempted to classify chicken ceca microbiome into enterotypes by determining whether these clusters were recapitulated in individual samples (Supplementary Fig. 6). Consistent with a recent study in humans, which suggests that enterotypes are an artefact of analysis,^10^ we found only a small fraction of samples captured all members of any one cluster. For example, only clusters 5 and 6 had at least 25% of their members present in more than 20% of the samples. This suggests that the cecal microbiomes are not readily classified into distinct enterotypes, but rather display considerable variability in taxonomic interactions.

### *Lactobacillus* has a polarizing effect on community composition

Therefore, instead of defining stable consortia through cluster memberships, we were interested in identifying keystone taxa in the cecal microbiome. The removal of species with a high number of interactions (hubs) has been known to significantly impact microbiome structure.^11^ Here, we extend this finding to form the hypothesis that the most influential taxa are likely to form many positive and negative associations with other taxa. We correlated the types of associations (positive or negative) of each taxon with its “hubness” (Fig. 2d). Remarkably, the vast majority of taxa displaying relatively large numbers of both negative and positive associations were *Lactobacilli*, suggesting a major influential role for this taxon in the cecal microbiome. This finding was consistent across studies for which *Lactobacillus* was present in 10% or more samples, i.e., studies based on sequencing V1-V3 or the V6-V8 regions of the 16S rRNA gene (Supplementary Figs 7, 8 and 9). While we showed above that cecal microbiomes are not readily classified into distinct enterotypes, the presence of *Lactobacilli* in clusters 5 and 6 may nonetheless help establish stable sub-clusters of taxa identified in a significant proportion of samples. For example, we note that at least 30% of the 1572 samples contain at least 25% of the members assigned to clusters 5 and 6 (Supplementary Fig. 6). Further, *Lactobacillus* dominates the most widely represented combinations of OTUs found across samples (Supplementary Table 3).

Despite experimental biases affecting our conclusions concerning the influence of different treatments on microbiome diversity, we find that *Lactobacilli* elicit a “Marmite effect” on other members of the cecal microbiome, so named after the British yeast-based spread known for producing a polarized “love/hate” reaction amongst consumers. This potential to influence community composition may partially explain the prominence of *Lactobacillus* strains as probiotics targeting foodborne infections.^12^ Through defining stable taxonomic associations, this study will help guide development of synthetic microbial consortia to promote gut health in chickens.

## Methods

### Collation of chicken cecal datasets

Survey sequence data from 19 chicken cecal studies published before 31 May 2017 were collated prior to meta-analysis to identify strains associated with healthy chickens (Supplementary table 1). Studies were identified through a systematic literature search using the terms “chicken cecal microbiome”, “chicken microbiome”, “chicken gut microbiome”, “broiler microbiome”, and “layer microbiome” on NCBI PubMed^13^ and Google Scholar, and the terms “poultry”, “chicken”, “broiler”, “layer” on the online server MGRAST.^14^ To be included in the meta-analysis, the study needed to be: (1) based on 16S rRNA survey sequence data (irrespective of hypervariable region used); (2) publicly accessible; and (3) have associated relevant metadata and sequence quality score information. Of 37 studies initially identified, only 19 passed the aforementioned criteria. All data were either found in the supplementary data of publications, the online server MGRAST,^14^ NCBI SRA,^13^ or the European Nucleotide Archive.^15^

### Processing of 16S rRNA gene sequences and data analysis

To maintain consistency across analyses, all datasets were processed using the QIIME package. For Illumina-generated datasets, paired-end reads were joined with *fastq-join* with an allowed maximum difference of 15 % and a minimum overlap of 35 bp. *Split_libraries_fastq.py* command truncated reads following three consecutive base calls with a Phred score of <20, and then discarded reads whose length were <75% of their original length following truncation. A custom script, suggested by QIIME developers (https://gist.github.com/walterst/ab88ae59a8900a2fa2da), was used to locate and truncate forward and reverse primers. For datasets generated by the 454 FLX Roche platform, fastq files were first converted to fasta and qual files, the *split_libraries.py* script removed primers, filtered out reads with homopolymer runs greater than 6, an average Phred score < 25, and read lengths outside of designated ranges. Appropriate read lengths for each dataset were based on expected read length of the hypervariable region being sequenced. Sequences were clustered into operational taxonomic units (OTUs) at 97 % similarity against the SILVA database^16^ (v128) using the *pick_closed_reference_otus.py* script from QIIME with reverse strand matching enabled. QIIME was also used to conduct microbial composition and diversity analyses. Alpha diversity was assessed using the Shannon and Chao1 index, beta diversity was assessed using the unweighted and weighted UniFrac distances. Non-parametric *t*-test was used for alpha diversity comparisons, *p*-values were adjusted for multiple correction using the Benjamini-Hochberg false discovery rate.^17^ Spearman correlations were computed between breeds using their taxonomic profiles, and breeds were clustered using the Cluster 3.0 software (the settings used were average linkage and correlation-centred).

### Co-occurrence network generation and clustering analysis

SparCC^18^ was used to generate all correlation networks. The correlation network with all samples included was built for OTUs that had more than 100 reads. Correlation networks for individual studies sequenced by Roche 454 were generated for OTUs that had more than 10 reads, while correlation networks for studies sequenced by Illumina MiSeq were generated for OTUs with more than 200 reads (Each Illumina MiSeq sample had approximately 20 times more coverage depth than Roche 454 samples). SparCC was run with 100 bootstraps to detect correlations between OTUs, correlations with *p*-values less than 0.05 were considered significant. To improve our understanding of relationships between co-occurring OTUs, ClusterONE^19^ was employed to cluster OTUs into groups on the basis of positive correlations of co-occurrence using default settings. The interaction score between two clusters was computed by taking the mean of all interaction scores between the OTUs within the two clusters. In the final clustered network, rare genera (defined as those with less than 15 OTUs) are grouped under higher level classifications (order or family) to reduce the complexity of the figure. The correlation network is visualized using the software Cytoscape (3.6.0).^20^ Clustered network, proportion stacked bar, charts and scatter plots where generated with matplotlib in Python 3.6. The code is available on the Parkinson Lab github account (https://github.com/ParkinsonLab/metaanalysis-chicken-ceca-paper). When identifying specific species for select OTUs, OTU sequences were compared against their own reference databases (e.g., *Lactobacillus* sequences were only aligned with other *Lactobacilli* sequences in NCBI’s non-redundant nucleotide collection database^13^ using BLAST.^21^ The *eclat* function from the arules R package^22^ was used to determine the combinations of OTUs that are present in the most samples.

## Electronic supplementary material

Supplementary Information

## Data Availability

All datasets analysed during the current study are included in the published articles listed in Supplementary Table 1.

## Electronic supplementary material

**Supplementary Information** accompanies the paper on the *npj Biofilms and Microbiomes* website (10.1038/s41522-018-0070-5).
