# Supplementary material for: Lactobacillus elicits a 'Marmite effect' on the chicken cecal microbiome
Source: NPJ Biofilms Microbiomes. 2018 Nov 9;4:27. doi: 10.1038/s41522-018-0070-5 (PMC6226495; doi:10.1038/s41522-018-0070-5)
Supplement: Supplementary file 1 — Supplementary Information [file 41522_2018_70_MOESM1_ESM.pdf]

Table S1. Sample and sequencing information for studies included in the meta-analysis.

PUBMED ID	Year	Experimental conditions	Breeds	Age	Number of sequences	Number of cecal samples	Pooled? (# of cecal samples/sequencing sample)	Sequencing depth (median)	Read length (bp)	Controls	Bead-beating step	DNA extraction	rRNA region	Sequencing platform
22114729[1]	2011	AGP	Ross	0, 7, 14,35	171,041	160	10	9,893	120-178	NA	Yes	QIAamp DNA stool mini kit	V3	GS FLX
24391931[2]	2013	Healthy	Cobb 500	25	635,507	209	No	1,404	300-600	NA	Yes	Repeated bead-beating + column (Yu and Morrison, 2004)	V1-V3	454 FLX
24657972[3]	2014	Healthy	Ross	42	548,553	40	No	4,192	350	NA	Yes	QIAamp DNA stool mini kit	V1-V3	454 FLX
25167074[4]	2014	Necrotic enteritis	Cobb 500	16	311,633	92	2	3,669	300-600	NA	Yes	Repeated bead-beating + column (Yu and Morrison, 2004)	V1-V3	454 FLX
25806087[5]	2015	Age and C. jejuni infection	Cobb 500	7, 14, 21, 42	1,799,440	64	16	419,334	2x150	NA	No	QIAamp DNA stool mini kit	V3	Illumina miseq
25887695[6]	2015	Healthy	Cobb 500	25	498,629	163	No	1,714	300-600	NA	Yes	Repeated bead-beating + column (Yu and Morrison, 2004)	V1-V3	454 FLX
26161743[7]	2015	Feed supplement and C.jejuni infection	Ross 308	35	114,762	30	No	2,857	100-250	NA	Yes	phenol-chloroform-isoamyl extraction	V2-V3	Ion Torrent
26425940[8]	2015	AGP	Cobb 500	44	958,143	120	No	3,220	200-600	NA	Yes	phenol-chloroform-isoamyl extraction	V6-V8	454 FLX
26835461[9]	2016	Vaccine and probiotics	White Leghor n	0,1,3, 7,14	4,008,379	130	No	24,821	2x150	Negative control	Yes	MO BIO Power Soil kit	V4	Illumina Miseq
26925052[10]	2016	Feed conversion ratio	Cobb 500	25	1,464,610	205	No	1,855	300-600	NA	Yes	Repeated bead-beating + column (Yu and Morrison, 2004)	V1-V3	454 FLX
27129897[11]	2016	Free range vs broiler	Broiler, free range	NA	3,477,532	84	No	26,967	NA	NA	No	QIAamp DNA stool mini kit	V3	Illumina miseq
27139888[12]	2016	Location	Tibetan	> 300 days old	275,089	105	15	16,976	2x300	NA	No	QIAamp DNA stool kit	V3-V4	Illumina miseq
27242676[13]	2016	Fresh vs reused litter	Broiler	10, 35 days	147,670	40	4	3,940	200-600	NA	Yes	QIAamp DNA stool kit	V1-V3	454 FLX
27921008[14]	2016	Age and C. jejuni infection	Ross 308	21, 28	2,110,924	30	No	40,559	NA	NA	No	MO BIO Power Soil kit	V3 -V5	Illumina Miseq
28066358[15]	2016	Performance	Ross 308	26	2,407,083	48	No	24,389	2x250	NA	No	FastDNA SPIN kit	V1-V2	Illumina Miseq
28222110[16]	2017	AGP	Cobb 500	43	5,694,695	93	No	43,893	2x250	NA	Yes	EZNA Stool DNA kit	V4	Illumina Miseq
28286717[17]	2017	Growth performance and feed	Cobb 500	25	446,732	91	No	1,843	300-600	NA	Yes	Repeated bead-beating + column (Yu and Morrison, 2004)	V1-V3	454 FLX
28243710[18]	2017	Growth performance and AGP	Cobb 500	25	628,094	191	No	1,622	300-600	NA	Yes	Repeated bead-beating + column (Yu and Morrison, 2004)	V1-V3	454 FLX
28367146[19]	2017	Feed supplement	Ross 308	35	7,230,853	55	No	73,019	2x250	Negative control	Yes	phenol-chloroform-isoamyl extraction	V4	Illumina Miseq

Table S2. T-test comparisons for boxplots displayed in Figure S4.

		Group1	Group2	Group1 mean	Group1 std	Group2 mean	Group2 std	t stat	p-value (FDR)
V1–V3	Shannon	Monensin, nicarbazin, bacitracin	None	2.43	0.70	3.13	0.64	-8.40	0.0001
	Chao1	Monensin, nicarbazin, bacitracin	None	28.94	10.65	44.34	17.00	-7.96	0.0001
V4	Shannon	Enramycin	Avilamycin	6.28	0.25	6.46	0.32	-1.69	1
		Halquinol	Avilamycin	6.23	0.51	6.46	0.32	-1.49	1
		Virginiamycin	Avilamycin	6.31	0.34	6.46	0.32	-1.23	1
		Halquinol	Virginiamycin	6.23	0.51	6.31	0.34	-0.49	0.87
		Halquinol	Enramycin	6.23	0.51	6.28	0.25	-0.37	0.841846
		None	Bacitracin	6.41	0.38	6.40	0.36	0.12	0.9021
		Bacitracin	Enramycin	6.40	0.36	6.28	0.25	1.01	0.692571
		Bacitracin	Avilamycin	6.40	0.36	6.46	0.32	-0.51	0.9216
		Halquinol	Bacitracin	6.23	0.51	6.40	0.36	-1.04	0.784
		Bacitracin	Virginiamycin	6.40	0.36	6.31	0.34	0.68	0.839667
		None	Enramycin	6.41	0.38	6.28	0.25	1.11	1
		None	Avilamycin	6.41	0.38	6.46	0.32	-0.37	0.9025
		Halquinol	None	6.23	0.51	6.41	0.38	-1.10	0.8592
		Enramycin	Virginiamycin	6.28	0.25	6.31	0.34	-0.23	0.87675
		None	Virginiamycin	6.41	0.38	6.31	0.34	0.77	0.839813
	Chao1	Enramycin	Avilamycin	753.76	71.08	816.59	56.19	-2.69	0.0495
		Halquinol	Avilamycin	786.87	79.10	816.59	56.19	-1.17	0.3738
		Virginiamycin	Avilamycin	815.51	52.58	816.59	56.19	-0.05	0.9606
		Halquinol	Virginiamycin	786.87	79.10	815.51	52.58	-1.13	0.364636
		Halquinol	Enramycin	786.87	79.10	753.76	71.08	1.19	0.400833
		None	Bacitracin	849.66	85.77	833.65	66.83	0.56	0.616714
		Bacitracin	Enramycin	833.65	66.83	753.76	71.08	3.17	0.02475
		Bacitracin	Avilamycin	833.65	66.83	816.59	56.19	0.76	0.520962
		Halquinol	Bacitracin	786.87	79.10	833.65	66.83	-1.72	0.23
		Bacitracin	Virginiamycin	833.65	66.83	815.51	52.58	0.81	0.5345
		None	Enramycin	849.66	85.77	753.76	71.08	3.29	0.042
		None	Avilamycin	849.66	85.77	816.59	56.19	1.24	0.409688
		Halquinol	None	786.87	79.10	849.66	85.77	-2.01	0.1674
		Enramycin	Virginiamycin	753.76	71.08	815.51	52.58	-2.65	0.0555
		None	Virginiamycin	849.66	85.77	815.51	52.58	1.27	0.465
V6 – V8	Shannon	Bacitracin	Virginiamycin	7.14	0.34	7.04	0.34	1.38	0.26835
		None	Bacitracin	7.20	0.32	7.14	0.34	0.80	0.4242
		None	Virginiamycin	7.20	0.32	7.04	0.34	2.23	0.0918
	Chao1	Bacitracin	Virginiamycin	632.50	60.04	612.09	55.03	1.57	0.3732
		None	Bacitracin	631.36	67.91	632.50	60.04	-0.08	0.9364
		None	Virginiamycin	631.36	67.91	612.09	55.03	1.38	0.2637

Table S3. Highly co-occurring OTUs. Table of the most popular combinations of OTUs and the number of samples they appear in. The OTUs are named according to the SILVA database, in brackets are shown their corresponding taxonomy. The combinations are numbered according to their popularity (number of samples they appear in).

Combination	OTUs in combination	Number of samples
1	GQ07075411388 (Lactobacillus),GQ13574111431 (Lactobacillus),KF84220311417 (Bacteroides)	355
2	GQ17545811439 (Anaerotruncus),GQ86425211491 (unclassified Lachnospiraceae),GU12817511435 (unclassified Ruminococcaceae)	314
3	GQ07075411388 (Lactobacillus),GQ13574111431 (Lactobacillus),HM30633011392 (Lactobacillus),KF84220311417 (Bacteroides)	306
4	DQ05738311395 (Lachnoclostridium),GQ17545811439 (Anaerotruncus),GQ86425211491 (unclassified Lachnospiraceae),GU12817511435 (unclassified Ruminococcaceae)	278
5	AF37622911349 (Butyricicoccus),GQ86425211491 (unclassified Lachnospiraceae),KF49452111281 ([Ruminococcus] torques group)	269
6	AF37622911349 (Butyricicoccus),FJ95069411472 (Escherichia-Shigella),GQ86425211491 (unclassified Lachnospiraceae),KF49452111281 ([Ruminococcus] torques group)	243
7	DQ05738311395 (Lachnoclostridium),GQ17537511452 (unclassified Ruminococcaceae),GQ17545811439 (Anaerotruncus),GQ86425211491 (unclassified Lachnospiraceae),GU12817511435 (unclassified Ruminococcaceae)	242
8	GQ07075411388 (Lactobacillus),GQ13574111431 (Lactobacillus),GQ26787611424 (Lactobacillus),HM30633011392 (Lactobacillus),KF84220311417 (Bacteroides)	240
9	AF37622911349 (Butyricicoccus),FJ95069411472 (Escherichia-Shigella),GQ86425211491 (unclassified Lachnospiraceae),HQ78789311439 (unclassified Ruminococcaceae),KF49452111281 ([Ruminococcus] torques group)	211
10	AB50636411521 (Ruminococcaceae UCG-005),GQ44847111385 (Subdoligranulum),HQ81505411440 (Sellimonas)	207

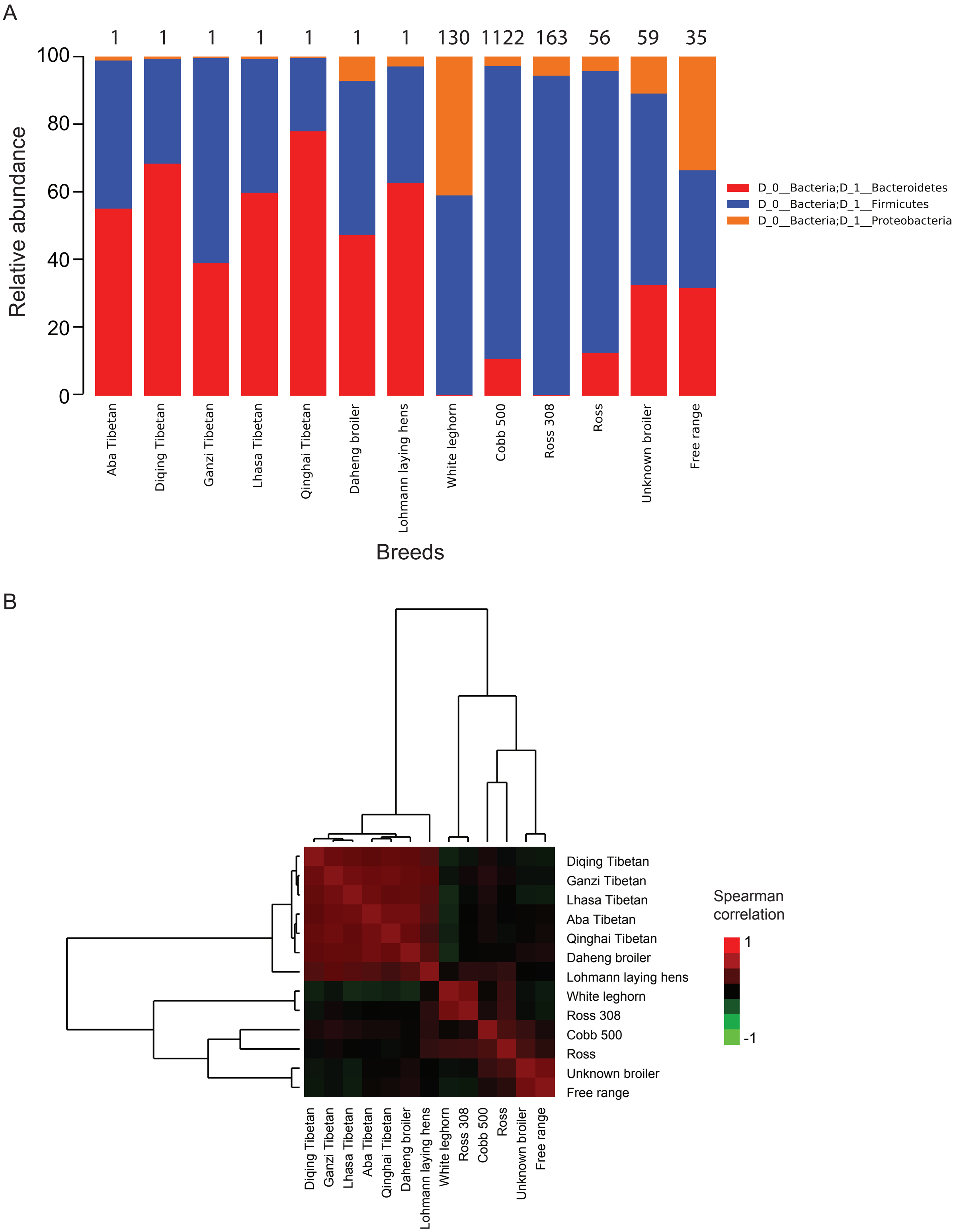

Figure S1. A) Relative abundance of the most abundant phyla by chicken breeds. Numbers on top of bars are the number of sequencing samples for each breed, note that certain samples are pooled from multiple chicken cecal samples (see supplemental table 1). Only taxa present at greater than 1 % were included. B) Clustering of breeds according to spearman correlations calculated from the taxonomic profiles of individual samples and heat map of breed correlations.

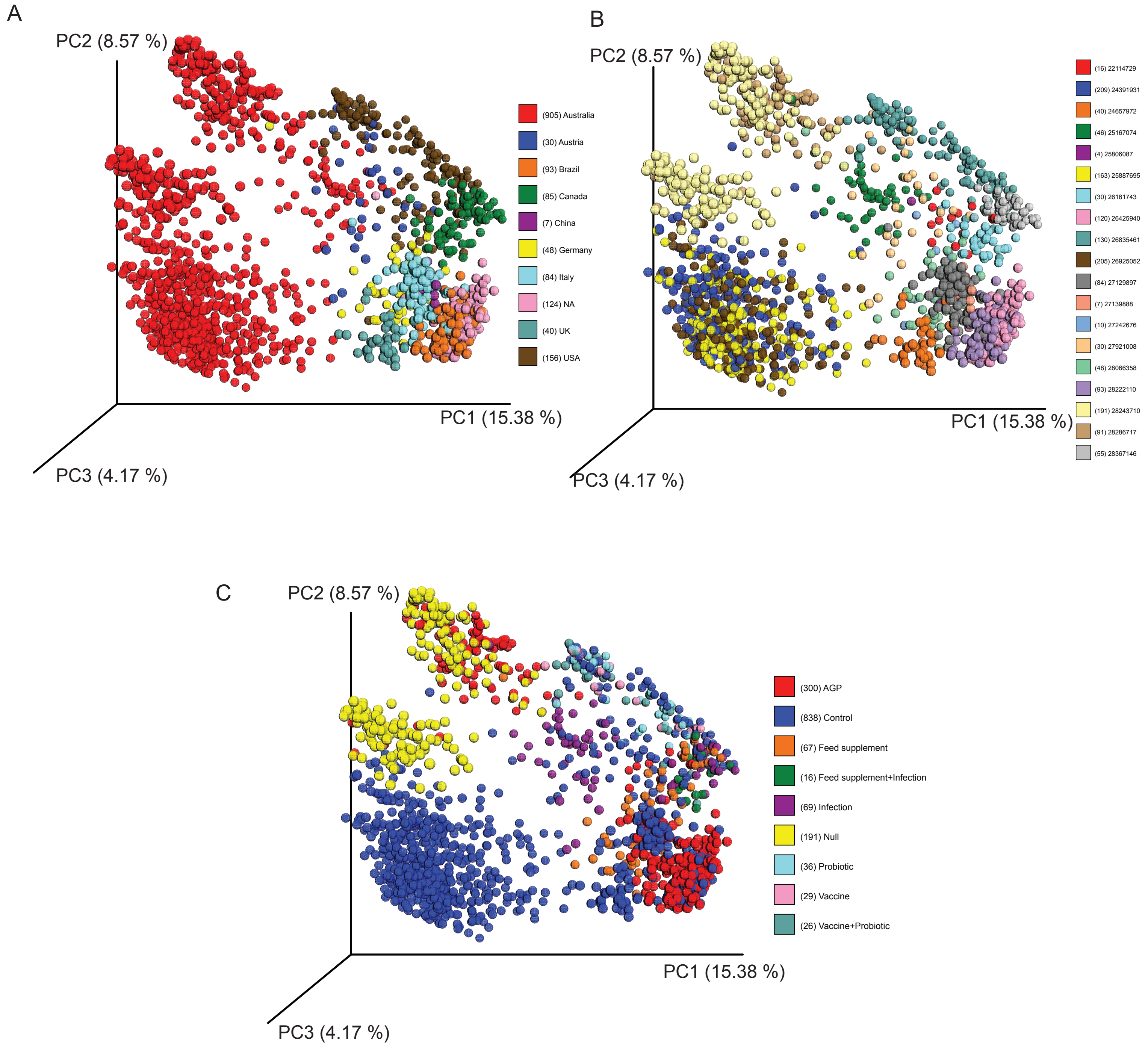

Figure S2. PCoA plots of unweighted UniFrac distances classified by A) country, B) study (PubMed ID), and C) Treatment. Numbers in brackets show the number of samples in each partition. The axes are labelled according to the amount of variation explained by each principal component (PC).

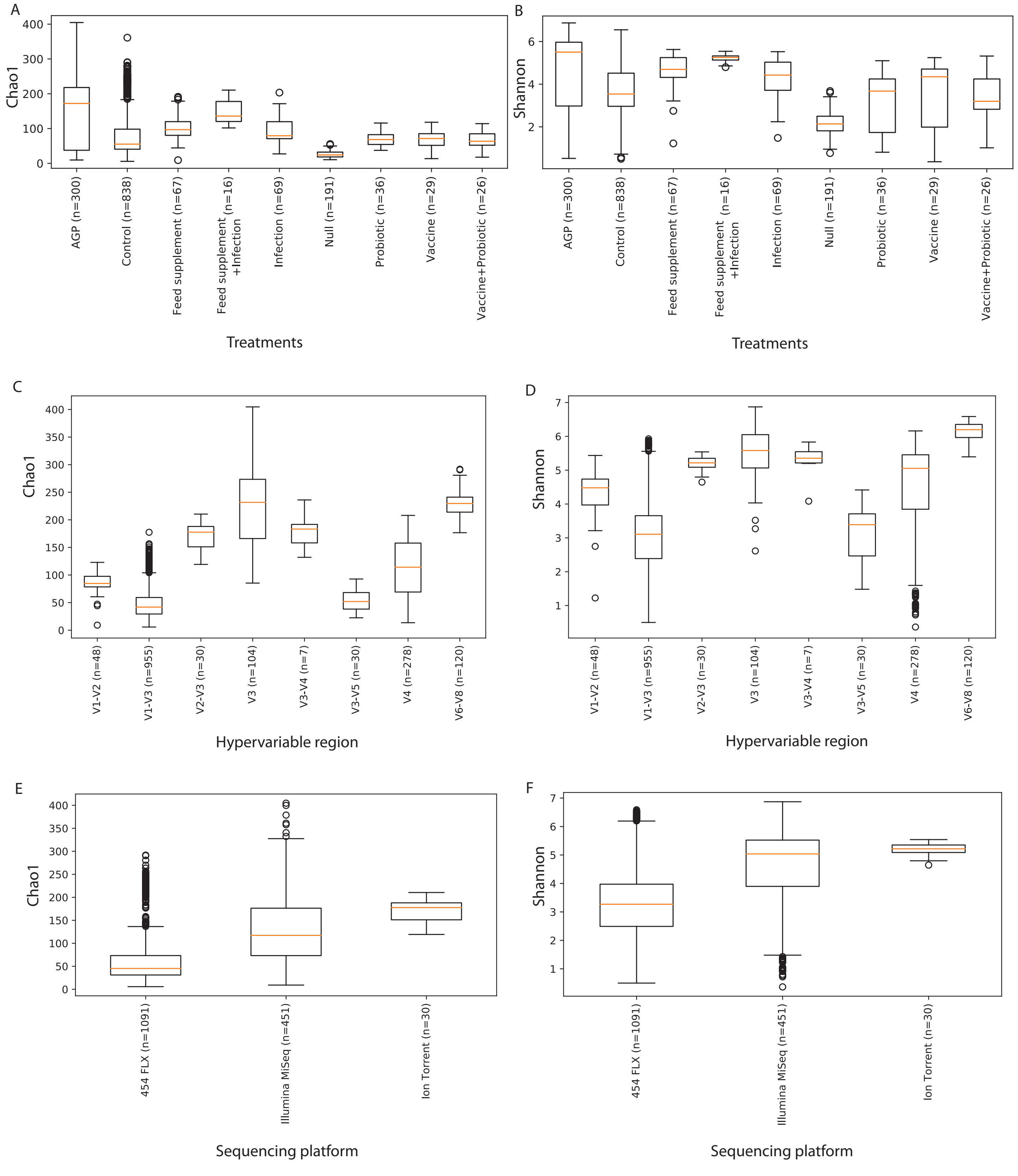

Figure S3. Differences in alpha diversity between treatments. Boxplot of alpha diversities for A) different treatments, measured with the Chao1 index, B) and the Shannon index C) different hypervariable regions, measured with the Chao index and D) the Shannon index, E) different sequencing platforms measured with the Chao1 index and F) the Shannon index. n represents the number of samples for each partition. Centre orange line marks the median, the bounds of the box represent the first and third quartiles, and the whiskers extend to 1.5 times the interquartile range.

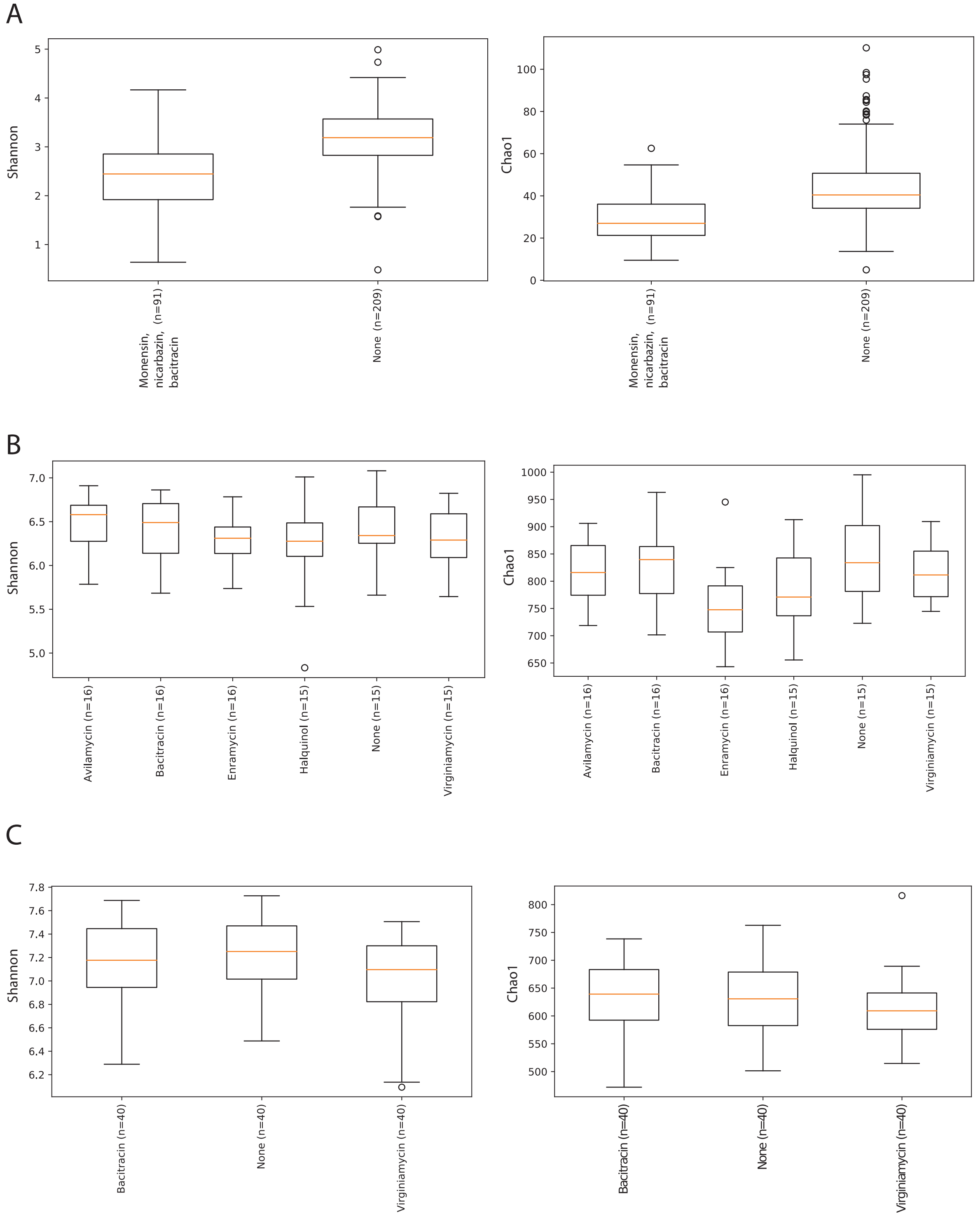

Figure S4. Differences in alpha diversity between AGPs and non-AGP treated samples using the Shannon and Chao1 metric for individual studies sequenced using different hypervariable region. A) Alpha diversity comparisons for samples sequenced using V1-V3 hypervariable region. Studies with PubMed IDs 24391931 (n=209) and 28286717 (n=91) were used as they were both conducted by the same research group that used the same chicken breeds and sampled at the same time point. B) Alpha diversity comparisons for samples sequenced from study 28222110 (V4 region). C) Alpha diversity comparisons for samples sequenced from study 26425940 (V6-V8 region). Centre orange line marks the median, the bounds of the box represent the first and third quartiles, and the whiskers extend to 1.5 times the interquartile range.

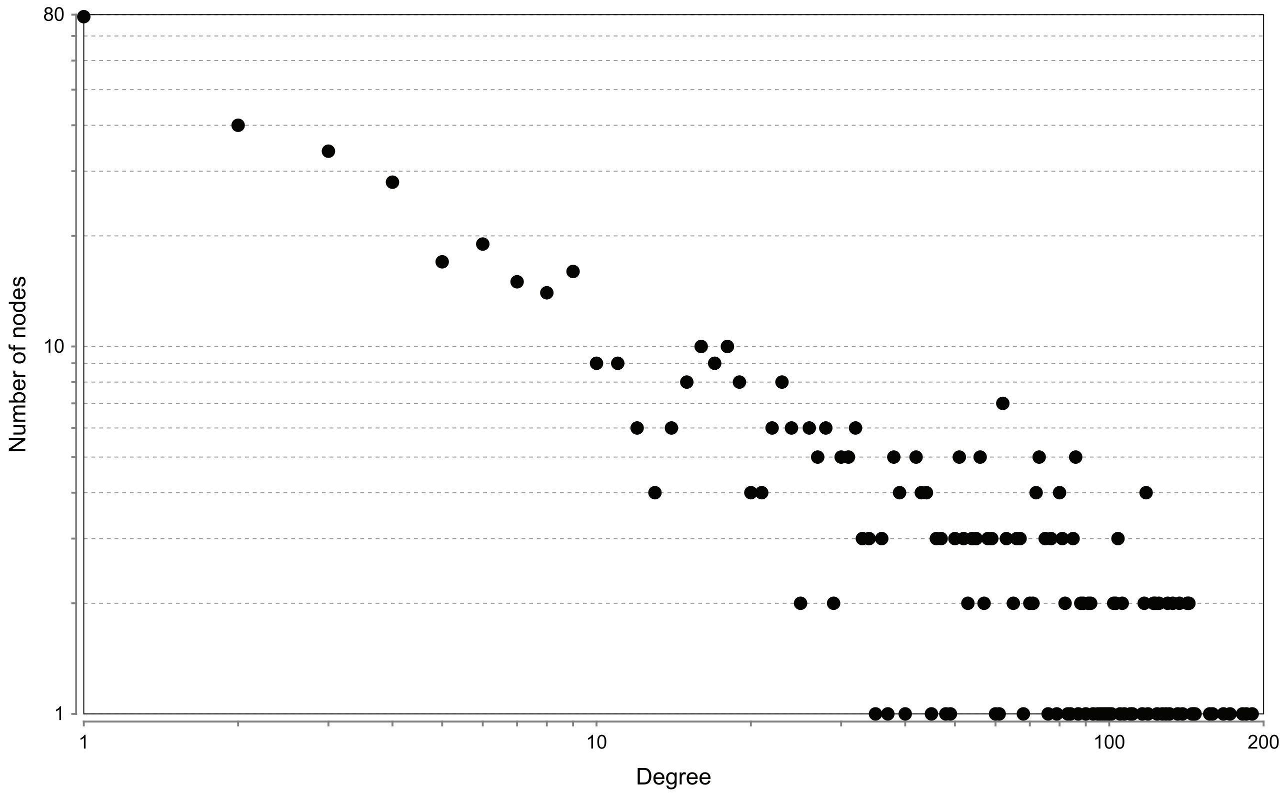

Figure S5. Log-log scatter plot of node degree (number of interactions for each OTU) versus number of nodes to describe OTU interactions distribution in Figure 2. The relationship between the two variables is linear, indicating that the distribution of the number of OTU interactions follows a power law.

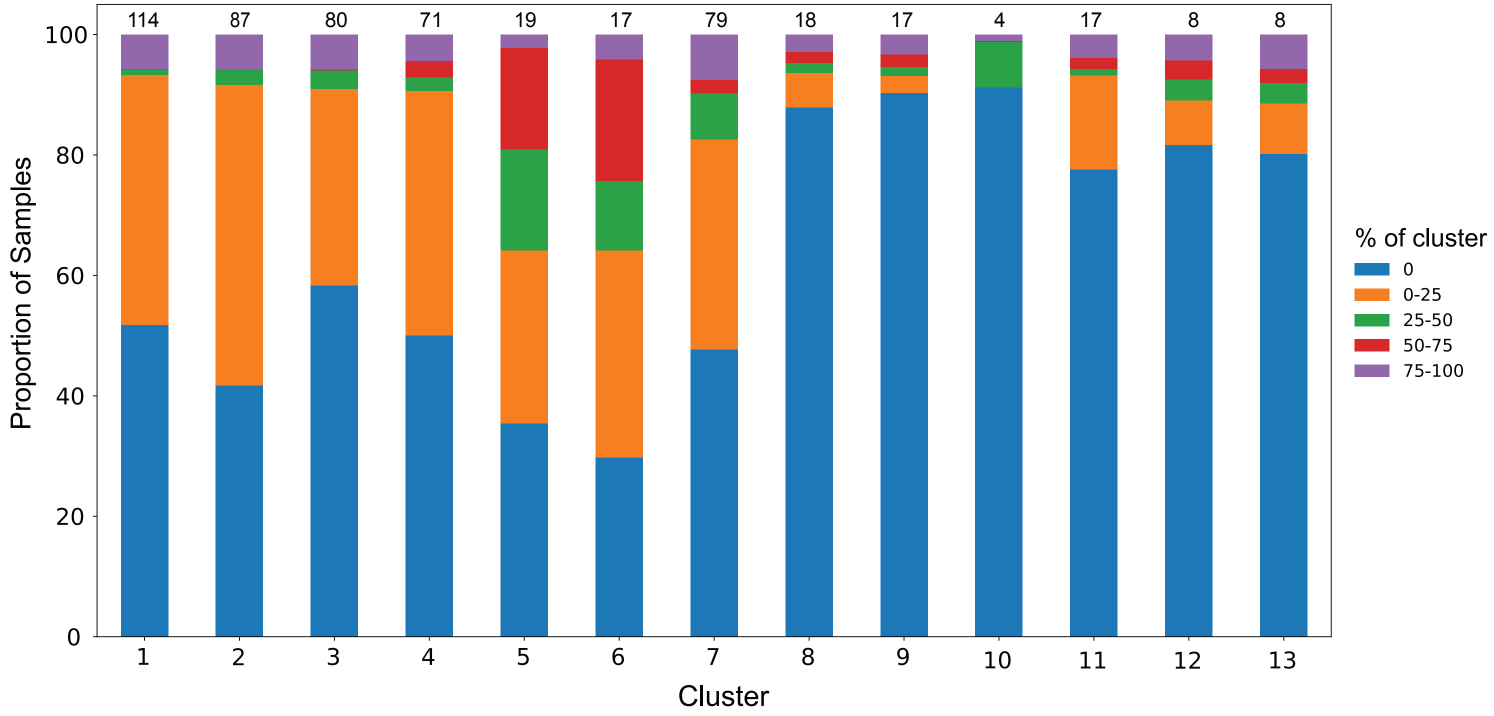

Figure S6. Consistency of co-occurring taxa clusters. Stacked bar chart showing how many samples each cluster can be found in, coloured according to proportion of clusters. Numbers shown on top of each bar are the number of OTUs in each cluster.

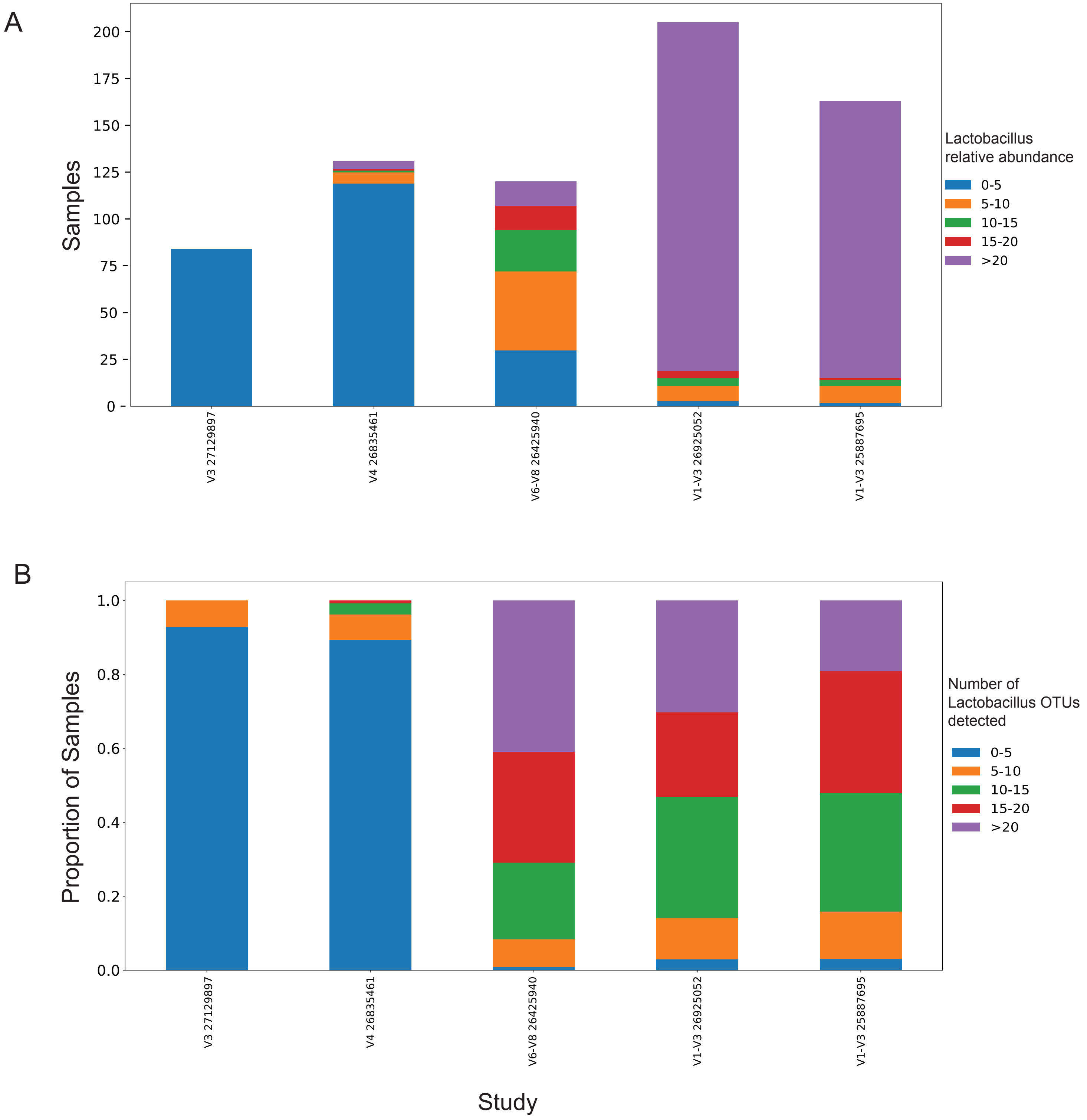

Figure S7. Stacked bar charts of *Lactobacillus* presence in different studies. Each stacked bar is labelled first with the hypervariable region used, followed by the PubMed ID of the study. A) Stacked bar chart showing number of samples with the relative abundance of *Lactobacillus* greater than the thresholds specified in the legend. B) Stacked bar chart showing proportion of samples that detected *Lactobacillus* OTUs at a number greater than the thresholds set in the legend. For the two studies that sequenced the V3 and V4 regions, a *Lactobacillus* OTU is only counted if it is covered by more than 20 reads in a sample, whereas all non-singleton OTUs are counted in a sample for the remaining 3 studies because they were sequenced by Roche 454 as opposed to Illumina MiSeq. Evaluating both graphs together shows that studies that sequenced V6-V8 and V1-V3 regions detected more *Lactobacillus* in most of their samples.

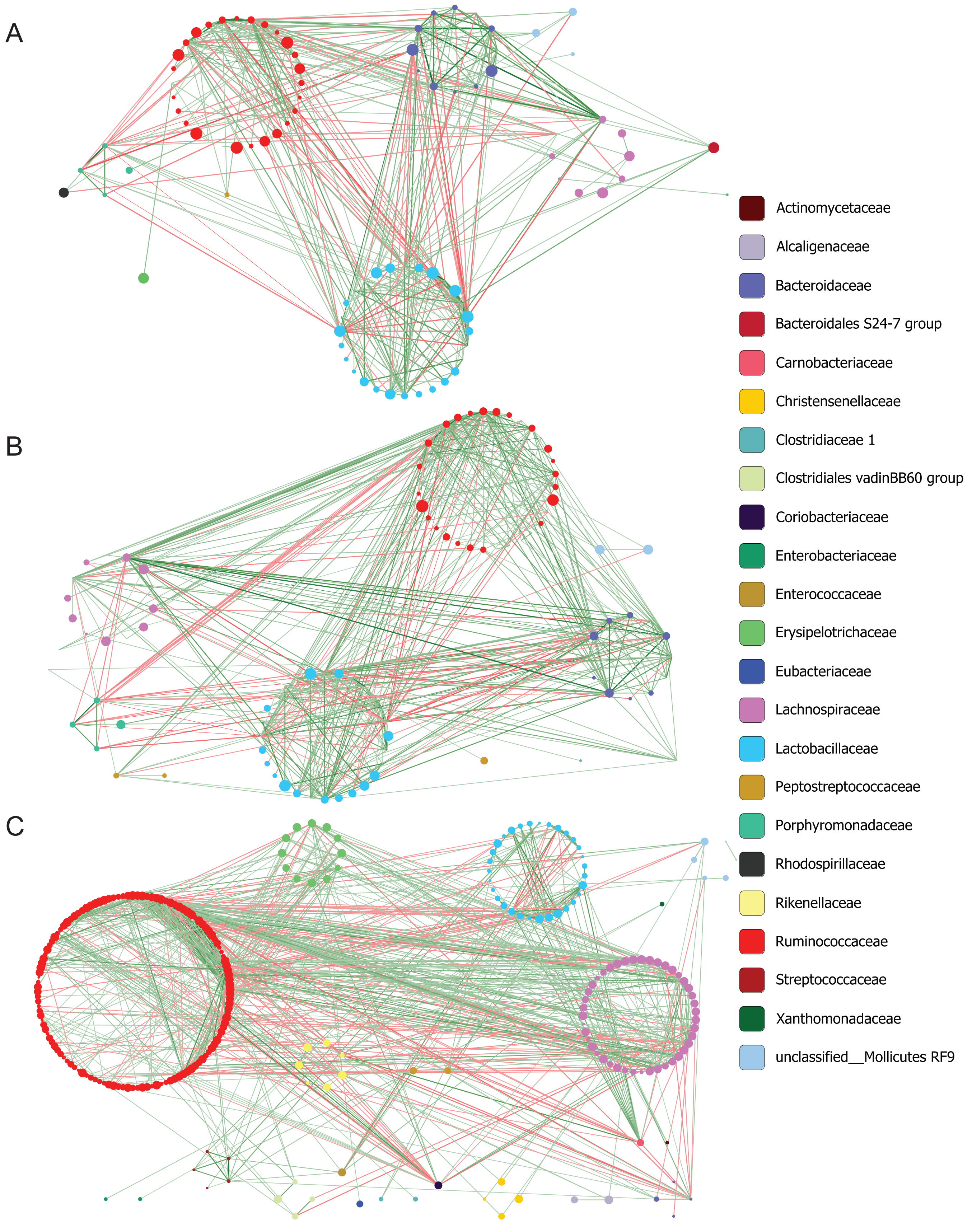

Figure S8. Co-occurrence networks generated using samples from individual studies A) Correlation network generated using samples from study 25887695 (V1-V3), B) 26925052 (V1-V3), and C) 26425940 (V6-V8). Networks were generated using OTUs with more than 10 reads. OTUs are grouped by family, node sizes represent the number of samples that contain those OTUs. Green edges are positive correlations and red edges are negative correlations, correlations with an absolute value smaller than 0.25 are not shown.

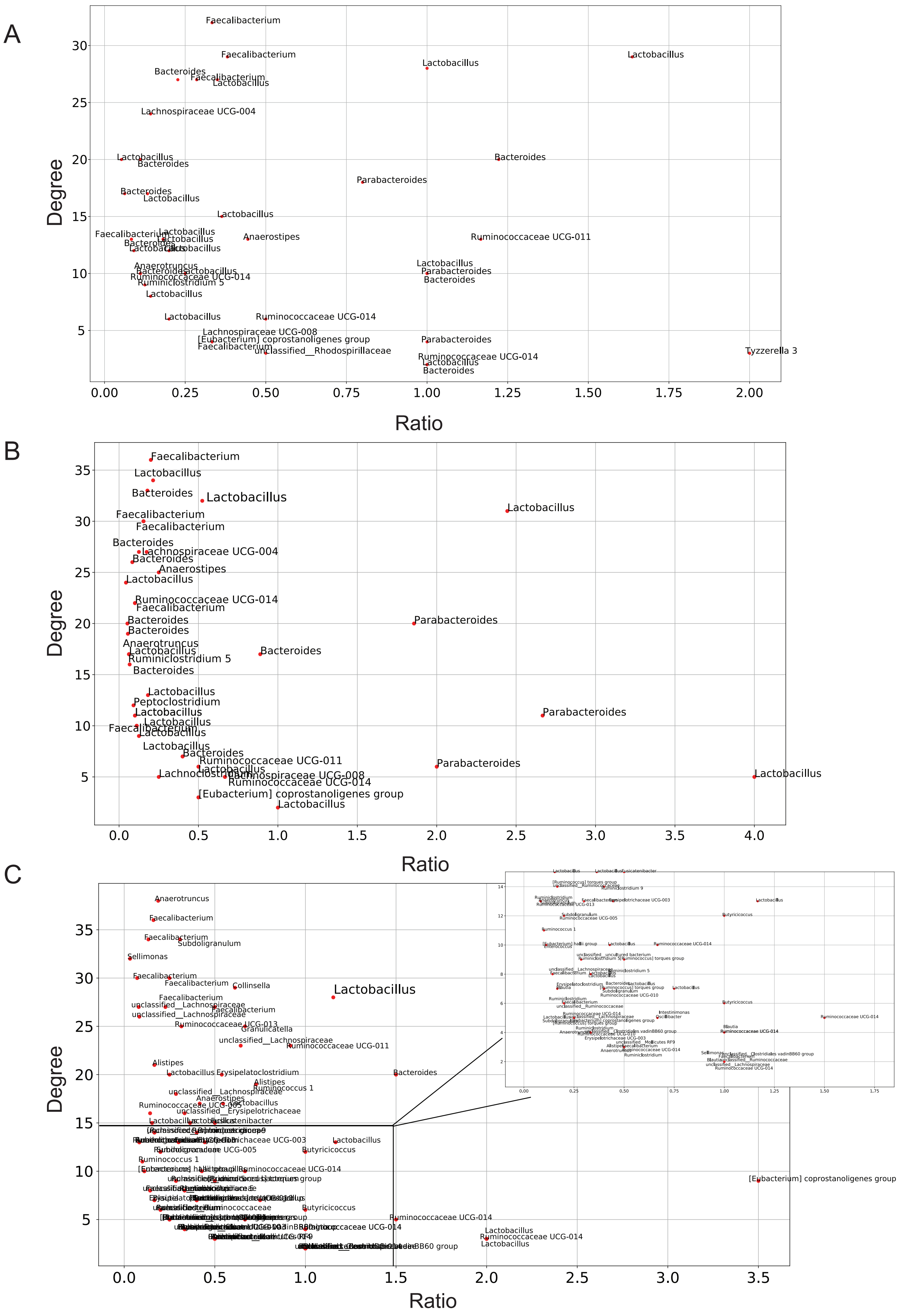

Figure S9. Scatter plots of ratio (negative:positive interactions) versus degree plot for correlation networks generated for 25887695 (V1-V3), B) 26925052 (V1-V3), and C) 26425940 (V6-V8).

Supplemental References

1. Danzeisen, J. L., Kim, H. B., Isaacson, R. E., Tu, Z. J. & Johnson, T. J. Modulations of the Chicken Cecal Microbiome and Metagenome in Response to Anticoccidial and Growth Promoter Treatment. *PLoS One* **6,** e27949 (2011).

2. Stanley, D., Geier, M. S., Hughes, R. J., Denman, S. E. & Moore, R. J. Highly Variable Microbiota Development in the Chicken Gastrointestinal Tract. *PLoS One* **8,** e84290 (2013).

3. Sergeant, M. J. *et al.* Extensive Microbial and Functional Diversity within the Chicken Cecal Microbiome. *PLoS One* **9,** e91941 (2014).

4. Stanley, D., Wu, S.-B., Rodgers, N., Swick, R. A. & Moore, R. J. Differential responses of cecal microbiota to fishmeal, Eimeria and Clostridium perfringens in a necrotic enteritis challenge model in chickens. *PLoS One* **9,** e104739 (2014).

5. Mohd Shaufi, M. A., Sieo, C. C., Chong, C. W., Gan, H. M. & Ho, Y. W. Deciphering chicken gut microbial dynamics based on high-throughput 16S rRNA metagenomics analyses. *Gut Pathog.* **7,** 4 (2015).

6. Stanley, D., Geier, M. S., Chen, H., Hughes, R. J. & Moore, R. J. Comparison of fecal and cecal microbiotas reveals qualitative similarities but quantitative differences. *BMC Microbiol.* **15,** 51 (2015).

7. Thibodeau, A. *et al.* Chicken Caecal Microbiome Modifications Induced by Campylobacter jejuni Colonization and by a Non-Antibiotic Feed Additive. *PLoS One* **10,** e0131978 (2015).

8. Neumann, A. P. & Suen, G. Differences in major bacterial populations in the intestines of mature broilers after feeding virginiamycin or bacitracin methylene disalicylate. *J. Appl. Microbiol.* **119,** 1515–1526 (2015).

9. Ballou, A. L. *et al.* Development of the Chick Microbiome: How Early Exposure Influences Future Microbial Diversity. *Front. Vet. Sci.* **3,** 2 (2016).

10. Stanley, D., Hughes, R. J., Geier, M. S. & Moore, R. J. Bacteria within the Gastrointestinal Tract Microbiota Correlated with Improved Growth and Feed Conversion: Challenges Presented for the Identification of Performance Enhancing Probiotic Bacteria. *Front. Microbiol.* **7,** 187 (2016).

11. Mancabelli, L. *et al.* Insights into the biodiversity of the gut microbiota of broiler chickens. *Environ. Microbiol.* **18,** 4727–4738 (2016).

12. Zhou, X. *et al.* Cecal microbiota of Tibetan Chickens from five geographic regions were determined by 16S rRNA sequencing. *Microbiologyopen* **5,** 753–762 (2016).

13. Wang, L., Lilburn, M. & Yu, Z. Intestinal Microbiota of Broiler Chickens As Affected by Litter Management Regimens. *Front. Microbiol.* **7,** (2016).

14. Awad, W. A. *et al.* Age-Related Differences in the Luminal and Mucosa-Associated Gut Microbiome of Broiler Chickens and Shifts Associated with Campylobacter jejuni Infection. *Front. Cell. Infect. Microbiol.* **6,** 154 (2016).

15. Borda-Molina, D., Vital, M., Sommerfeld, V., Rodehutscord, M. & Camarinha-Silva, A. Insights into Broilers' Gut Microbiota Fed with Phosphorus, Calcium, and Phytase Supplemented Diets. *Front. Microbiol.* **7,** (2016).

16. Costa, M. C. *et al.* Different antibiotic growth promoters induce specific changes in the cecal microbiota membership of broiler chicken. *PLoS One* **12,** e0171642 (2017).

17. Crisol-Martínez, E., Stanley, D., Geier, M. S., Hughes, R. J. & Moore, R. J. Sorghum and wheat differentially affect caecal microbiota and associated performance characteristics of meat chickens. *PeerJ* **5,** e3071 (2017).

18. Crisol-Martínez, E., Stanley, D., Geier, M. S., Hughes, R. J. & Moore, R. J. Understanding the mechanisms of zinc bacitracin and avilamycin on animal production: linking gut microbiota and growth performance in chickens. *Appl. Microbiol. Biotechnol.* **101,** 4547–4559 (2017).

19. Thibodeau, A., Letellier, A., Yergeau, É., Larrivière-Gauthier, G. & Fravalo, P. Lack of Evidence That Selenium-Yeast Improves Chicken Health and Modulates the Caecal Microbiota in the Context of Colonization by Campylobacter jejuni. *Front. Microbiol.* **8,** 451 (2017).
